# Public perceptions of adult ADHD: Indications of stigma?

**DOI:** 10.1007/s00702-020-02279-8

**Published:** 2020-11-25

**Authors:** Emmet Godfrey, Anselm B. M. Fuermaier, Lara Tucha, Marah Butzbach, Matthias Weisbrod, Steffen Aschenbrenner, Oliver Tucha

**Affiliations:** 1 Faculty of Behavioral and Social Sciences, Department of Clinical and Developmental Neuropsychology, University of Groningen, Groningen, The Netherlands; 2 Department of Psychiatry and Psychotherapy, University Medical Center Rostock, Rostock, Gehlsheimer Str. 20, 18147 Rostock, Germany; 3 Department of Psychiatry and Psychotherapy, SRH Clinic Karlsbad-Langensteinbach, Karlsbad, Germany; 4 Center of Psychosocial Medicine, Department of General Psychiatry, University of Heidelberg, Heidelberg, Germany; 5 Clinical Psychology and Neuropsychology, SRH Clinic Karlsbad-Langensteinbach, Karlsbad, Germany

**Keywords:** Adult ADHD, Stigma, CAARS, WFIRS

## Abstract

Stigmatization represents a major barrier to treatment seeking across mental disorders. Despite this, stigma research on individual mental disorders remains in its infancy. Attention-deficit hyperactivity disorder (ADHD) in adults also represents an under-researched area—being far less studied than its child counterpart. This study examined the current state of public perceptions towards adult ADHD. A simulation group consisting of 105 participants performed the Weiss Functional Impairment Rating Scale (WFIRS) and Conners’ Adult ADHD Rating Scales (CAARS) as though they had ADHD. These scores were compared to a group consisting of 98 individuals with adult ADHD and a group of 117 healthy individuals both groups being instructed to complete the WFIRS and CAARS to the best of their abilities. Simulators were found to overestimate impairments in adult ADHD (to a large effect) in the domains of hyperactivity, DSM-IV hyperactivity-impulsivity, DSM-IV total, work, school, (to a medium effect) in family and social, and (to a negligible-small effect) in inattention, impulsivity, DSM-IV inattention, and life skills when compared to the ADHD group, and in all domains (to a large effect) when compared to the control group. Current and retrospective ADHD symptoms were found to be associated with more accurate perceptions in a number of domains. Evidence for the presence of perceptions considered to be stigmatizing was found, with largest effects present in the domains of hyperactivity, impulsivity, impairments at work, school, and engagement in risky behaviour.

## Introduction

Stigma, as defined by Goffman (1963), is a characterization of an individual which conveys a social identity that is devalued in a given social context (Meza, Monroy, Ma, & Mendoza-Denton). Stigmatization of mental disorders has and continues to be a primary concern in the field of mental health (Link 1987; Hinshaw and Cicchetti 2000; Lebowitz 2016). Gulliver et al. (2010) highlighted in their systematic review that stigma and embarrassment towards one’s own mental health problems is a major barrier for treatment seeking in young people (Masuch et al. 2019). Over the recent decades, the knowledge of researchers, mental health professionals, and the general public has seen a tremendous expansion in relation to mental health (Schomerus et al. 2012). Despite this apparent burgeoning in public knowledge, stigma towards mental disorders persists. Schomerus et al. (2012) showed in a systematic review and meta-analysis that while literacy concerning mental disorders had increased significantly over a period of 20 years, attitudes towards individuals with mental disorders have not become more positive, in the case of schizophrenia, they had even become more negative. Given that one of the most common ways of reducing stigma is to increase knowledge, this calls into question the relationship between knowledge of a mental disorder and stigmatization, and how the public perceive mental disorders to impact daily functioning (Kosyluck et al. 2016).

Stigma as a concept can be divided in many ways, such as public stigma, courtesy stigma, and self-stigma (Mueller et al. 2012). Stigma can come from others (public stigma), come from oneself (self-stigma), and affect others associated with the mental disorder (courtesy stigma). Corrigan and Shapiro (2010) conceptualized that stereotypes and prejudice were the cognitive expression of stigma, with discrimination being the behavioural expression. Not only does stigma exacerbates already existing symptoms of a mental illness, perceived stigma can prevent an individual from seeking treatment to help alleviate symptoms. Indeed, stigmatization can affect many facets of an individual’s life, such as education, social, housing prospects, healthcare, and employment (Lebowitz 2016). A study by Hipes et al. (2016) elegantly demonstrated this stigmatization in employment. They showed using fictitious job applications that individuals with a history of mental illness received significantly less call-backs than individuals without a history of mental illness but possessing a history of physical injury. Given the neurodevelopmental nature of ADHD, these negative perceptions pose a continuous threat from a multitude of different sources. While public stigma towards mental illness as a general concept is well researched, research on disorder-specific stigmatization remains in its early stages (Masuch et al. 2019).

Attention-deficit hyperactivity disorder (ADHD) is a neurodevelopmental disorder characterized by persistent periods of hyperactivity, impulsivity, and inattention that is pervasive across situations and leads to interference in daily functioning (Biederman 2005; Polanczyk et al. 2014). ADHD is one of the most common mental illnesses, with an estimated prevalence of 3–12% in children and 2–6% in adults (Canu et al. 2008). Children displaying ADHD symptoms will continue to experience symptoms into adolescence at a chance of 70–80% with a further likelihood of 50–70% of them meeting the diagnostic criteria in adulthood (Meza et al. 2019). Though the more overt symptoms of hyperactivity/impulsivity tend to dissipate upon adulthood, the more covert symptoms of inattention tend to persist (Biederman 2005). Despite potential changes in symptoms from childhood to adulthood, research has shown that stigmatization remains a constant presence (Biederman 2005; Mueller et al. 2006; Meza et al. 2019). Until recently, the majority of research into stigmatization of mental disorders has focused on psychotic disorders, depression, and mental illness as a whole (Masuch et al. 2019). In the cases when ADHD stigma was investigated, it overwhelmingly focuses on childhood ADHD (Mueller et al. 2012; Lebowitz 2016; Masuch et al. 2019). Despite this paucity of research, the presence of public stigma towards adult ADHD has been demonstrated (Lebowitz 2016; Masuch et al. 2019).

Individuals with ADHD find themselves stigmatized from many angles in their lives (Corrigan and Shapiro, 2010). Public stigma derives from large-scale population opinions towards individuals from groups perceived to be different, whether it be racially, behaviourally, cognitively, etc. (Mueller et al. 2012). This type of stigma is particularly destructive, as it can affect the groups directly, or can serve to increase other forms of stigma, such as self-stigma and courtesy stigma (Meza et al. 2019). Perceptions form quickly, Pelham and Bender (1982) showed that an increased desire for social distance away from children exhibiting ADHD-related behaviour in play settings can form as quickly as 30 min after contact. Commonly, aspects of ADHD symptomology are perceived as impoliteness, character weakness, immaturity, emotional dysfunctionality, and unreliability (Masuch et al. 2019). While there is compelling research into stigmatization as evidenced by desire for increased social distance, there is little research on public perceptions of how individuals with adult ADHD experience functional impairments (Lebowitz 2016).

Stigmatization is commonly combatted through education (Rüsch et al. 2005; Morgan et al. 2018). Two frequently used ways of informing people about mental illness are by contact and education (Kosyluk et al. 2016). Kosyluk and colleagues (2016) compared the efficacy of these two methods of intervention for reducing stigma in adult ADHD. Contact-based intervention used personal stories from individuals with the mental disorder to convey information, whereas the education-based approach employed a more information heavy presentation addressing myths and popular beliefs surrounding ADHD. Both approaches were found to reduce stigma; however, no difference between the effectiveness of either approaches was found. Kosyluk and colleagues (2016) theorized that contact and education-based interventions work by reducing social distance, as well as stigmatizing attitudes, and increasing positive beliefs about empowerment and treatment seeking. Though the two approaches differ in their execution, one commonality they share is that they seek to increase knowledge about the mental disorder and its symptomology to reduce stigma. It should follow that increased knowledge of ADHD would lead to more accurate perceptions of the disorder, and subsequently less stigmatization.

Ray and Hinnant (2009) conducted a literature review investigating how ADHD has been depicted in the media. They give a sense of the public view towards ADHD, their findings highlight that the media generally portrays the symptoms of ADHD using a classroom setting, and as such focus on the learning difficulties associated with ADHD. This could lead to an underrepresentation of the adult side of ADHD, which may lead the public to associate ADHD symptoms with those characteristic of childhood ADHD. They also found frequent references to the dangerousness of an individual with ADHD, both for themselves and for others. As such, the domains of daily life of an individual with ADHD which may be at particular risk of public stigma could be: inattention, hyperactivity, impulsivity, school, family, and engagement in risky behaviour.

The measurement of stigmatization and prejudice has often been compared, due in part to their similar nature in having covert and overt manifestations (Stuber et al. 2008). Indeed, prejudice and stereotypes even form the cognitive aspect of public stigma posited by Corrigan and Shapiro (2010). It has been a trend in research that overt expressions of prejudice have been reducing in frequency as time progresses (Stuber et al. 2008). The same cannot be said for more covert or “unconscious” expressions of prejudice, which have not shown the same reduction over time. The reduction in overt expressions of prejudice may be due to changes in political correctness which frowns upon public expressions of prejudice. The majority of research focuses on overt expressions of stigma (Stuber et al. 2008). Previous research has often used explicit methods to measure overt expressions of stigma. Commonly used methods to directly measure public stigma can be seen in the form of self-report questionnaires (Meza et al. 2019; Speerforck et al. 2019), behaviourally measuring desire for social distance from individuals with adult ADHD (Canu et al. 2008; Meza et al. 2019), and having participants respond to vignettes (Lebowitz 2016). Given the lack of research on covert expressions of public stigma, coupled with its apparent prevalence within society, it seems pertinent to use more implicit methods to measure stigmatization on a more unconscious level (Schomerus et al. 2012). This study proposes a new method to measure public perceptions at an implicit level. It aims to do this by instructing participants to feign ADHD and perform two questionnaires commonly used in the assessment of ADHD. In keeping with the Corrigan et al. (2010) conceptualization of stigma, this study aims to measure the cognitive aspect of public stigma by measuring participants’ perceptions towards ADHD. The advantage of this method is that it may give insight into how participants perceive individuals with adult ADHD to perform in a variety of domains, rather than a more general opinion concerning those with adult ADHD. Additionally, this method keeps the participant blind to the true purpose of the study, protecting against any potential social desirability bias (Fisher 1993; Gray 2002). This study represents not just a new approach to measuring perceptions associated to public stigma in adult ADHD, but an approach potentially applicable to measuring disorder-specific perceptions across mental disorders.

This study aimed to investigate how the public perceive adult ADHD, in which functional domains are perceptions least accurate, and whether ADHD knowledge or ADHD symptomology are associated with more accurate perceptions. Healthy individuals were assigned to either a simulation or control condition. Adult ADHD-diagnosed individuals were assigned to an ADHD group. Participants in the simulation group were instructed to complete the CAARS and WFIRS while pretending to be affected by ADHD; the control group was instructed to complete the same questionnaires to the best of their abilities; participants in the ADHD group were instructed to complete the same questionnaires to the best of their abilities. The accuracy of the simulation group’s estimations of impairments when compared to the ADHD groups’ estimations provides the measure for perception accuracy. Additionally, this study examined the relationship between knowledge of ADHD and perception accuracy. Finally, it investigated the relationship between ADHD symptomology and perception accuracy—that is, an individual’s proximity to ADHD symptoms without having a diagnosis of ADHD. Data used to measure the resilience of the CAARS (Fuermaier et al. 2016; Fuermaier et al. 2018) and WFIRS (Fuermaier et al. 2018) against feigned ADHD were repurposed for this study.

This leads to the following hypotheses: (1) Participants in the simulation group overestimate the extent of impairments in adult ADHD, particularly in the domains of inattention, hyperactivity, impulsivity, school, family, and risk (2) ADHD knowledge is negatively correlated with perception accuracy, suggesting that greater knowledge of ADHD is associated with more accurate perceptions, and (3) ADHD symptomology is negatively correlated with perception accuracy, suggesting that self-proximity to ADHD symptoms is associated with more accurate perceptions.

## Methods

### Patients with ADHD

One-hundred and thirty-seven individuals were considered for inclusion in the study. Ten participants did not meet the diagnostic criteria for a diagnosis of ADHD, and were excluded from the study. A total of 29 participants were excluded from the ADHD sample due to non-credible performance on the Test of Malingered Memory (TOMM; Tombaugh 1997) (10 participants) and the Groningen Effort Test (GET; Fuermaier et al. 2017) (19 participants). This left a total of 98 patients included in the study. The subtypes of ADHD found in the ADHD group were: 52 with combined, 45 with inattentive, and 1 with hyperactive-impulsive. They were either referred or self-referred by local psychiatrists or neurologists to the Departments of Psychiatry and Psychotherapy of the SRH Clinic Karlsbad-Lagensteinbach in Germany. All participants were offered a diagnostic assessment of ADHD and participation in the research project. The researchers informed the participants that the research project and the clinical assessment were isolated entities; it was made explicitly clear to participants that participation in the study was separate from the clinical diagnostic assessment. Participation was voluntary and no reward or compensation was given. Written informed consent was obtained from all participants prior to assessment. The diagnostic assessments for adult ADHD were administered by licensed, experienced, psychologists in accordance with the 4th edition of the Diagnostic and Statistical Manual of Mental Disorders (DSM-IV; American Psychological Association, 1994) criteria for ADHD including current symptoms using the ADHD Self-Report Scale (ASRS; Adler et al. 2006) and retrospective childhood symptoms using the Wender Utah Rating Scale-Short (WURS-K; Ward et al. 1993). This diagnosis of adult ADHD was the result of multiple sources of evidence (e.g. school reports, academic performance, occupational performance etc.) obtained from multiple places (e.g. employers, family members etc.).

### Healthy individuals

Two-hundred-and-thirty-three healthy individuals were considered for this study. One-hundred-and-seventeen of these healthy individuals were randomly assigned to the control group. One-hundred-and-sixteen were randomly assigned to the simulation group. Eleven participants were excluded from the simulation group based on insufficient scores on an item measuring their perceived effort (Effort Scale). This left a total of 222 healthy individuals included in the study (Control group *n* = 117; Simulation group *n* = 105). This sample was gathered in the context of a larger research project, which randomly assigned healthy individuals to one of several groups (Fuermaier et al. 2016, 2018). For the purpose of this study, only the control group and naïve simulation group were examined. Characteristics of each group are listed in Table 1. Recruitment was achieved using word of mouth, public announcements, and through associates of the researchers of the study. Informed written consent was obtained from all participants in the simulation and control groups. Additionally, none of the participants were taking a medication known to affect the central nervous system.

**Table 1:** Means and Standard Deviations of characteristics for Control, Simulation, and ADHD Groups

Variable	Control	Simulation	ADHD	F/Chi Squared Test	*p*
*N*	117	105	98		
Age (in years)	27.75 ± 10.88^a^	27.46 ± 10.87^a^	34.81 ± 11.10	*F*(2, 319) = 14.84	*p* < .01
Gender (male/female)	43/74	47/58	63/35	*χ*^*2*^(2) = 1.73	*p* = .42
Education (in years)	16.18 ± 2.68^a^	16.40 ± 4.75^a^	13.03 ± 3.95	*F*(2, 319) = 24.36	*p* < .01
IQ (Vocabulary skills)	101.44 ± 11.73	100.45 ± 10.77	104.45 ± 12.20	*F*(2, 297) = 3.09	*p* = .05
ADHD Knowledge	15.13 ± 6.73	16.09 ± 6.50	N/A	*F*(1, 214) = 1.12	*p* = .29
ASRS (Current Symptoms)	10.79 ± 6.07^a^	10.85 ± 6.13^a^	31.77 ± 9.31	*F*(2, 319) = 284.89	*p* < .01
WURS-K (Childhood Symptoms)	13.30 ± 10.14^a^	14.50 ± 10.35^a^	39.03 ± 14.43	*F*(2, 319) = 157.77	*p* < .01

*ADHD* attention deficit hyperactivity disorder, *IQ* Intelligence Quotient, *ASRS* ADHD Self Report Scale, *WURS-K* Wender Utah Rating Scale-Short version

Pairwise comparisons of all groups:

^a^Significant difference from ADHD group at alpha level of .05

To investigate demographic differences between the groups, a one-way analysis of variance (ANOVA) was performed taking GROUP (ADHD group; simulation group; control group) by AGE (in years), IQ (Multiple Choice Vocabulary Test), YEARS OF EDUCATION, RETROSPECTIVE ADHD SYMPTOMS (WURS-K), CURRENT ADHD SYMPTOMS (ASRS), and ADHD KNOWLEDGE (ADHD Knowledge Questionnaire). A Chi-squared test checked for differences on gender by taking GROUP (ADHD group; simulation group; control group) on GENDER. Any significant differences were then investigated further by pairwise comparisons using Tukey’s honest significance difference (Tukey’s HSD). Significance level was set to 0.05.

Comparisons of group characteristics are presented in Table 1. Age (in years) and education (in years) were found to be significantly different between the groups. The ADHD group had a significantly higher mean age and a significantly lower mean education (in years) when compared to the control and simulation groups. The groups did not differ significantly in regards to gender or IQ. Additionally, the control and simulation groups did not differ in respect to ADHD knowledge. In line with diagnostic expectations, the ADHD group had significantly higher scores for childhood symptoms (WURS-K) and current symptoms of ADHD (ASRS) when compared to both the control and simulation groups.

#### Materials

### Intellectual functions (vocabulary skills)

Intellectual functioning was estimated using the Multiple Choice Vocabulary Test (MWT-B; Lehrl 1995). This questionnaire can be used to give an estimate of general intelligence, particularly focused on crystallized intelligence. Consisting of 37 items, it involves selecting the correct word from multiple choices. The number of correct items of a participant is then compared to a representative sample which gives the sum score as an intelligence quotient.

### Wender utah rating scale

Past childhood ADHD symptom severity was assessed using the WURS-K (Ward et al. 1993). This questionnaire ascertains the participants’ ADHD symptomology when they were experiencing childhood. It consists of 25 items (Retz-Junginger et al. 2003). Participants respond to items on a 5-point scale ranging from 0 (being “does not apply”) to 4 (being “strongly agree”). These sum scores then give a retrospective impression of ADHD symptomology, with a higher sum score representing more endorsement of past ADHD symptomology.

### ADHD self-report scale

Current ADHD symptoms of participants were assessed with the ASRS (Adler et al. 2006). This questionnaire consists of 18 items related to ADHD symptomology as outlined by the DSM-IV. Participants responded to statements on a scale from 0 (being “never”) to 4 (being “very often”). A participant’s sum score was their total score on the questionnaire, with a higher sum score indicating greater current ADHD symptoms experienced by the participant.

### ADHD knowledge questionnaire

The knowledge of ADHD of participants in the control and simulation groups was assessed using an ADHD knowledge questionnaire. This questionnaire consisted of 34 statements regarding ADHD and was developed by Gaastra, Groen, Fuermaier, Tucha, and Tucha (2015). The items of the questionnaire measure knowledge across 4 domains: Symptoms (11 items), Diagnosis & Prevalence (10 items), Etiology (6 items), and Treatment (7 items). The combined score from these domains then gives an indication of ADHD specific knowledge. Responses were given as either “true”, “false”, or “do not know”. Sum scores ranged from 0 to 34, with 0 indicating no correct responses, and 34 indicating all responses correct.

### Symptom validity and compliance to test instructions

To ensure symptom validity and acceptable levels of effort, 3 effort measures were used: the TOMM (Tombaugh 1997), the GET (Fuermaier et al. 2017), and the Effort Scale (Fuermaier et al. 2016, 2018). The TOMM is a visual recognition test which aims to discriminate between malingered and actual memory impairments (Tombaugh 1997). The GET was designed to identify non-credible attention performance as a possible indication of feigned cognitive impairments (Fuermaier et al. 2017). Participants in the ADHD group completed either the TOMM or the GET. For the simulation group, one item examined how much effort the individual perceived they used to feign ADHD, and this was referred to as the Effort Scale (Fuermaier et al. 2016, 2018). Participants were asked “How much effort did you put into feigning ADHD?” Possible answers were on a scale from 1 to 5 (1 being “not at all” and 5 being “totally”). A score of < 4 was taken as sufficient evidence to exclude this participant.

### Weiss functional impairment rating scale self-report

The WFIRS-S is a questionnaire aimed at estimating a participant’s ability to function in 7 domains (CADDRA 2017). It is primarily used in the assessment of ADHD for both adults and children. It allows for the assessment of emotional, behavioural, and cognitive functioning in domains clinically relevant to ADHD. The domains are Family (8 items), Work (11 items), School (11 items), Life Skills (12 items), Self-Concept (5 items), Social (9 items), and Risk (14 items), resulting in a total of 70 items. Items are framed as statements (e.g. “having problems with family”) and the participants indicated how closely the statement related to them. Responses are given on a Likert scale ranging from 0 (being “Never or Not at All”) and 3 (being “Very Often or Very Much”), and included the option to choose “not applicable”. The participant’s sum score in a domain was calculated by taking their score on all items in that domain and dividing it by the number of items endorsed in that domain.

### Conners’ adult ADHD rating scales

The Conners’ Adult ADHD Rating Scales is a questionnaire designed to assess the severity of ADHD symptoms in adults (Conners et al. 1998). The assessment helps clinicians ascertain which domains associated with ADHD symptoms are being affected. The questionnaire consists of 9 subscales, Inattention/Memory Problems (12 items), Hyperactivity/Restlessness (12 items), Impulsivity/Emotional Lability (12 items), Problems with Self-Concept (6 items), and additionally, three subscales assess ADHD symptoms as outlined by the DSM-IV. These are Inattentive Symptoms (9 items), Hyperactive-Impulsive Symptoms (9 items), plus an aggregated Total ADHD Symptoms. Additionally, there is the ADHD Index (12 items), which distinguishes between ADHD adults and nonclinical adults. Finally, the Inconsistency Index identifies random or careless responding. Certain item scores contributed to more than one subscale. Participants responded to statements with how much the statement applied to them. Responses were given on a 4-point Likert scale ranging from 0 (being “not at all/never”) and 3 (being “very much/very frequently”). Sum scores were calculated for each participant by finding the total score for each subscale.

## Design and procedure

### ADHD group

Assessment was conducted individually and in a quiet environment. Participants were instructed to complete the CAARS and WFIRS to the best of their ability. It was made clear to them not to ask for help from the examiner or to discuss their responses. Completion of the CAARS and WFIRS was done in the context of a larger study which in total took approximately 2.5 h. All participants were debriefed. The research was conducted in compliance with ethical standards and approved by the local institutional ethical committee.

### Simulation and control groups

Assessment took place individually and in a quiet environment. A number of demographics were first obtained from the participants (age, gender, years of education, occupation etc.). Current and retrospective ADHD symptoms were then assessed using the WURS-K and the ASRS. Participants were asked whether they had a history of psychiatric or neurological diseases or pharmacological treatment. Following this, a short presentation was shown to the simulation group only, outlining the potential benefits of a diagnosis of adult ADHD. This presentation intentionally did not include information regarding the nature of adult ADHD and its symptomology. Participants in the simulation group were instructed to complete the CAARS and WFIRS questionnaires as though they had adult ADHD. To help ensure that they feigned ADHD, they were told that the participant who feigned most convincingly would win a recent tablet personal computer, and overdoing symptoms would decrease their chances of winning. Control group participants were instructed to complete the CAARS and WFIRS questionnaires to the best of their abilities. Both groups were told not to seek help from the examiner. The Effort Scale was then presented to the simulation group to ascertain how much effort they put into feigning ADHD. Upon completion, participants were given a debriefing. Assessment took approximately 30–60 min. The research was conducted in compliance with ethical standards and approved by the local institutional ethical committee.

### Statistical analysis

#### Group comparisons

Due to violations of normality and homogeneity of variance, it was decided to use nonparametric tests. All analyses were performed using the statistical software package SPSS 25.0. Five participants were removed from the analysis of the WFIRS scores due to administrative errors. These 5 participants were deemed appropriate for inclusion in all analyses, not including those WFIRS scores. An initial alpha level of 0.05 was set for all analyses, but was adjusted for multiple comparisons if applicable. To examine the difference in CAARS subscales, and the difference in WFIRS subscales, between the ADHD group, the simulation group, and the control group, Mann–Whitney *U* tests were performed. These compared the pairs of GROUP (ADHD group; simulation group; control group) by CAARS INATTENTION, CAARS HYPERACTIVITY, CAARS IMPULSIVITY, CAARS SELF-CONCEPT, CAARS DSM-IV INATTENTION, CAARS DSM-IV HYPERACTIVITY-IMPULSIVITY, and CAARS DSM-IV TOTAL. Following this, each pair of GROUP (ADHD group; simulation group; control group) was then compared on WFIRS FAMILY, WFIRS WORK, WFIRS SCHOOL, WFIRS LIFE SKILLS, WFIRS SELF-CONCEPT, WFIRS SOCIAL, and WFIRS RISK. A Bonferroni adjustment was administered (0.05/7 = 0.007) to account for multiple testing and Cohen’s d was calculated for each comparison to determine the size of the differences between groups. Interpretation of the magnitude of Cohen’s *d* was in accordance with Cohen (1988), with *d* < 0.20 representing a negligible effect, 0.20 ≤ *d* < 0.50 a small effect, 0.50 ≤ *d* < 0.80 a medium effect, *d* ≥ 0.80 a large effect.

### Correlational analysis

**Fig. 1:**
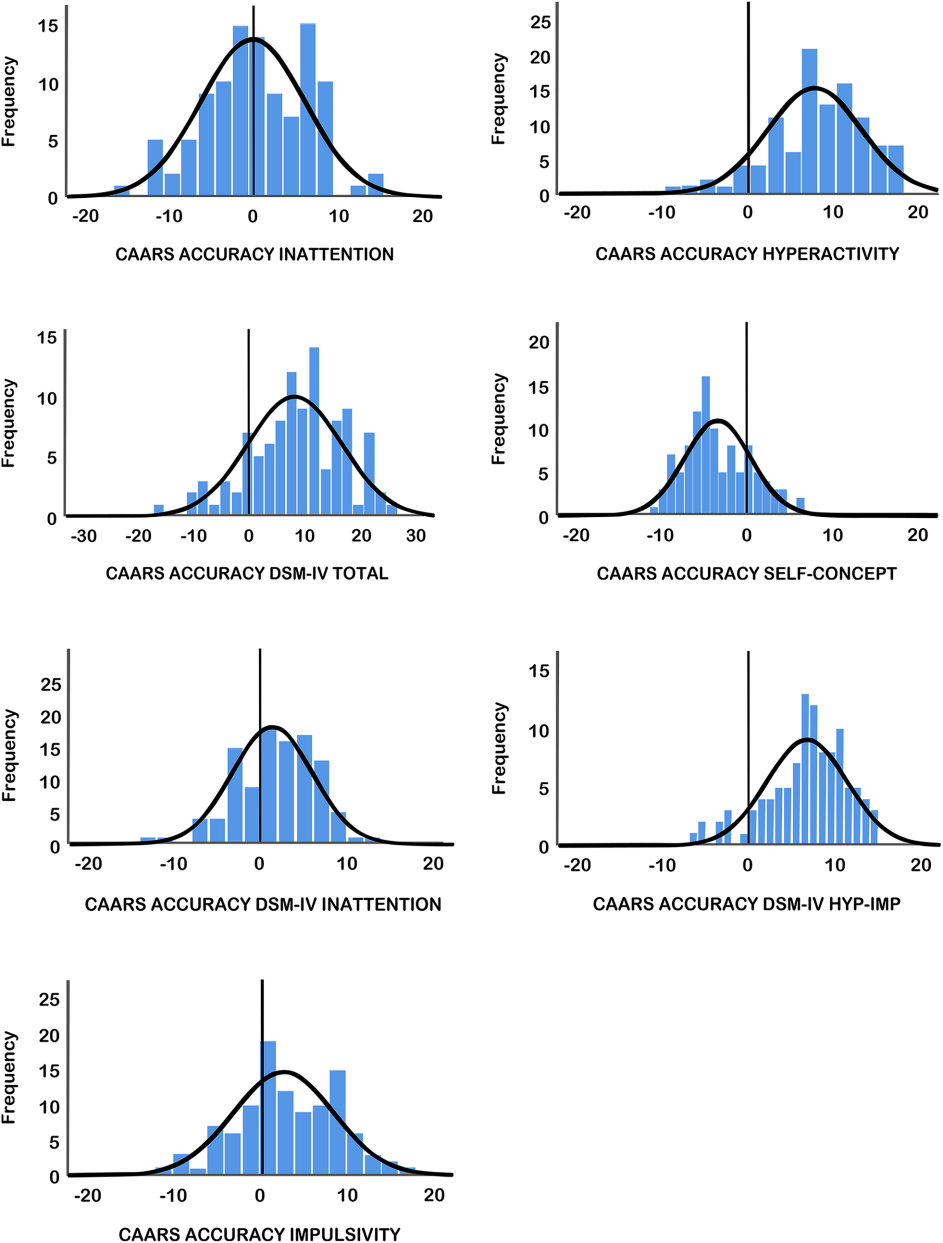
Estimation accuracy of simulation group on the Conners’ Adult ADHD Rating Scale. Estimation accuracy variables were calculated by subtracting the mean CAARS scores of the ADHD group from the individual scores of the simulation group. Scores above 0 are indications of overestimation of impairments by the simulation group

**Fig. 2:**
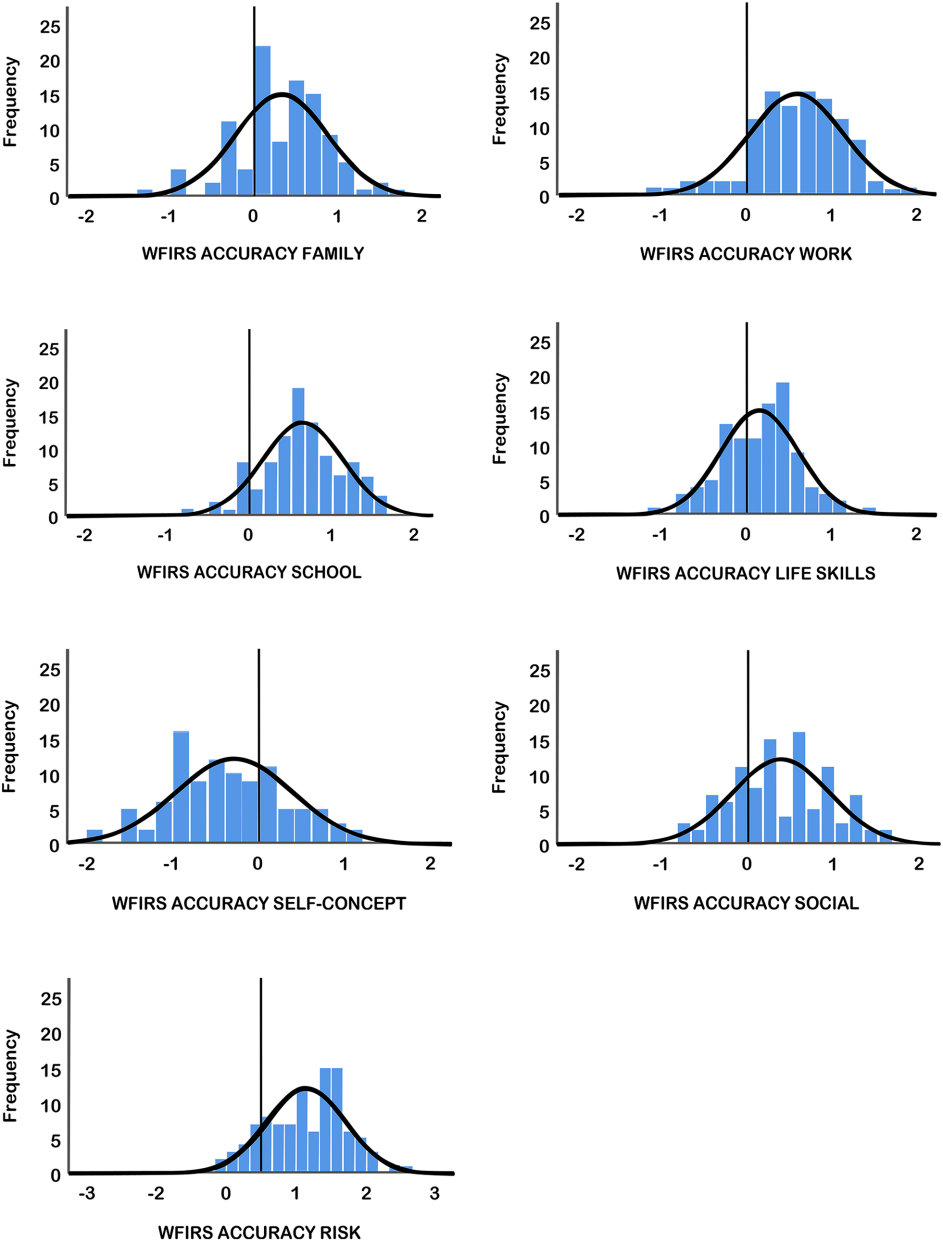
Estimation accuracy of simulation group on Weiss Functional Impairment Rating Scale subscales. Estimation accuracy variables were calculated by subtracting the mean WFIRS scores of the ADHD group from the individual scores of the simulation group. Scores above 0 are indications of overestimation of impairments by the simulation group

To derive a variable of perception accuracy of the simulation group, the mean CAARS and WFIRS scores of the ADHD patient group were subtracted by the individual scores of the simulation group. These new difference scores (see Figs. 1 and 2) were then taken as estimates of accuracy for the simulation group—referred to as ACCURACY variables. As this study was specifically interested in perceptions relating to stigma, difference scores below or equal to 0 (indicating an exact or positive perception of ADHD) were excluded from the correlational analysis—as scores larger than 0 indicate negative beliefs towards ADHD (which can be interpreted as potential evidence of stigmatization).

A correlational analysis was used to examine the relationship between ADHD KNOWLEDGE, CURRENT ADHD SYMPTOMS, RETROSPECTIVE ADHD SYMPTOMS, AGE, IQ, YEARS OF EDUCATION, and POSITIVE ACCURACY ESTIMATIONS (accuracy estimations > 0; indicating negative perceptions) of the WFIRS subscales and CAARS subscales for the simulation group. Given the violations of normality and homogeneity of variance in the data, Spearman’s rho was taken as the nonparametric measure for correlation. Interpretation of the size of the correlation was in accordance to the categorization of Cohen (1988), with *r* < 0.10 representing a negligible effect, 0.10 ≤ *r* < 0.30 a small effect, 0.30 ≤ *r* < 0.50 a medium effect, and *r* ≥ 0.50 a large effect. Although this analysis involves multiple testing, it was decided not to use the Bonferroni adjustment, as it was deemed too rigid for this more exploratory analysis. As such, the focus will be on the effect sizes found, though references will be made to their significance. This correlational analysis took IQ, RETROSPECTIVE ADHD SYMPTOMS, CURRENT ADHD SYMPTOMS, ADHD KNOWLEDGE, YEARS OF EDUCATION, and AGE, and correlated them with all of the CAARS ACCURACY and WFIRS ACCURACY variables in the simulation group.

## Results

### Simulation group vs. ADHD group as a measure of perceptions

Figure 1 shows the distribution of simulators’ estimations of impairments in ADHD across the CAARS subscales. Scores above 0 represent overestimation, whereas scores below 0 represent underestimation of impairments. Each domain in Fig. 1 shows a tendency to be larger than 0, except for SELF-CONCEPT. This can be taken as an indication for overestimation of impairments in all domains, save for SELF-CONCEPT. In Fig. 2, the distribution of simulators’ estimations of impairments in ADHD across the WFIRS subscales is displayed. As with Figs. 1, 2 shows overestimation of impairments in all domains except for SELF-CONCEPT.

The means and standard deviations of each group across the CAARS and WFIRS subscales are seen in Table 2. The test statistics and effect sizes of the Mann–Whitney *U* test taking GROUP (ADHD group; simulation group) by all CAARS variables are given in Table 3. Positive effect sizes represent an overestimation of impairments by the simulation group, with negative effect sizes representing underestimation. Simulators had significantly higher scores than the ADHD group on CAARS HYPERACTIVITY, CAARS DSM-IV HYPERACTIVITY-IMPULSIVITY, and CAARS DSM-IV TOTAL. All of these differences represented large positive effect sizes (*d* ≥ 0.8). Simulators had significantly lower scores than the ADHD group on CAARS SELF-CONCEPT to a large negative effect. No significant difference was found between the groups on CAARS INATTENTION, CAARS IMPULSIVITY, or CAARS DSM-IV INATTENTION. Nevertheless, the effect sizes here represented small positive effects (0.20 ≤ *d* < 0.50) for all except CAARS INATTENTION, which had a negligible negative effect. The simulation group showed significantly higher scores than the ADHD group on WFIRS FAMILY, WFIRS WORK, WFIRS SCHOOL, WFIRS SOCIAL, and WFIRS RISK. These effect sizes represent medium-to-large positive effects (0.50 ≤ *d* < 0.80). This effect was not found for WFIRS LIFE SKILLS, however, the effect size represented a small positive effect. Simulators had significantly lower scores than the ADHD group on WFIRS SELF-CONCEPT to small negative effect size.

**Table 2:** Means, Standard Deviations, and Mann–Whitney *U* statistics for ADHD, Simulation, and Control Group across the CAARS and WFIRS subscales

Variable	M ± SD of ADHD	M ± SD of Simulation	M ± SD of Control	(Z, p) of Simulation vs ADHD	(Z, p) of ADHD vs. Control	(Z, p) of Simulation vs. Control
CAARS Inattention	22.69 ± 6.13	22.48 ± 6.13	9.70 ± 5.55	− 0.29, 0.77	− 10.84, < 0.001*	− 11.08 < 0.001*
CAARS Hyperactivity	18.79 ± 7.26	26.53 ± 5.50	10.03 ± 6.57	− 7.47, < 0.001*	− 8.05, < .0.001*	− 11.66 < 0.001*
CAARS Impulsivity	20.23 ± 7.79	22.87 ± 5.73	8.45 ± 5.40	− 2.41, 0.016	− 9.88, < .0.001*	− 11.73 < 0.001*
CAARS Self-concept	11.85 ± 4.41	8.36 ± 3.86	5.26 ± 3.38	− 5.50, < 0.001*	− 9.33, < 0..001*	− 5.91 < 0.001*
CAARS DSM-IV Inattention	18.09 ± 4.39	19.30 ± 4.63	6.51 ± 4.71	− 1.89, 0.058	− 11.28, < 0.001*	− 11.70 < 0.001*
CAARS DSM-IV Hyp-Imp	12.53 ± 5.58	19.21 ± 4.66	5.68 ± 4.89	− 7.92, < 0.001*	− 8.45, < 0.001*	− 11.81 < 0.001*
CAARS DSM-IV Total	30.46 ± 8.43	38.50 ± 8.47	12.20 ± 9.04	− 6.39, < 0.001*	− 10.81, < 0.001*	− 11.94 < 0.001*
WFIRS Family	1.34 ± 0.65	1.66 ± 0.54	0.51 ± 0.44	− 3.75, < 0.001*	− 8.93, < 0.001*	− 11.17, < 0.001*
WFIRS Work	1.16 ± 0.68	1.73 ± 0.55	0.42 ± 0.49	− 6.27, < 0.001*	− 8.05, < 0.001*	− 11.14, < 0.001*
WFIRS School	1.28 ± 0.80	1.92 ± 0.49	0.56 ± 0.52	− 5.69, < 0.001*	− 6.11, < 0.001*	− 11.44, < 0.001*
WFIRS Life skills	1.40 ± 0.62	1.54 ± 0.45	0.65 ± 0.43	− 1.82, 0.069	− 8.61, < .0.001*	− 10.64, < .0.001*
WFIRS Self-concept	1.82 ± 0.82	1.53 ± 0.67	0.67 ± 0.58	− 2.91, 0.004*	− 9.05, < 0.001*	− 8.59, < 0.001*
WFIRS Social	1.24 ± 0.65	1.62 ± 0.56	0.43 ± 0.41	− 3.99, < 0.001*	− 9.02, < .0.001*	− 11.58, < 0.001*
WFIRS Risk	0.78 ± 0.52	1.42 ± 0.57	0.50 ± 0.39	− 7.31, < 0.001*	− 4.59, < .0.001*	− 10.25, < .0.001*

*CAARS* Conners’ Adult ADHD Rating Scales, *WFIRS* Weiss Functional Impairment Rating Scale. *indicating significant difference at the Bonferroni adjusted level of 0.007, *M* mean, *SD* standard deviation, *Z* z-score, *p* *p*-value, *ADHD* = attention-deficit hyperactivity disorder, *DSM-IV* = Diagnostic and Statistical Manual of Mental Disorders 4th Edition, *Hyp-Imp* = hyperactivity-impulsivity

**Table 3:** Effect Sizes (Cohen’s d) of group differences across CAARS and WFIRS subscales

Variable	Simulation vs. ADHD	ADHD vs. control	Simulation vs control
CAARS Inattention	− 0.04	3.26*	2.23*
CAARS Hyperactivity	1.45*	1.57*	2.52*
CAARS Impulsivity	0.35	1.35*	2.56*
CAARS Self-concept	− 0.91*	2.15*	0.86*
CAARS DSM-IV Inattention	0.27	3.76*	2.53*
CAARS DSM-IV Hyp-Imp	1.61*	1.73*	2.60*
CAARS DSM-IV Total	1.12*	3.23*	2.68*
WFIRS Family	0.58*	1.98*	2.33*
WFIRS Work	1.12*	1.61*	2.34*
WFIRS School	0.99*	1.06*	2.52*
WFIRS Life skills	0.26	1.83*	2.69*
WFIRS Self-concept	− 0.43*	2.04*	1.44*
WFIRS Social	0.62*	2.02*	2.54*
WFIRS Risk	1.43*	0.70*	1.95*

For the comparison Simulation vs ADHD, a positive effect size indicates overestimation, and a negative effect size indicates underestimation

*ADHD* attention-deficit hyperactivity disorder, * indicating significant difference at the Bonferroni adjusted level of 0.007, *CAARS* Conner’s Adult ADHD Rating Scales, *WFIRS* Weiss Functional Impairment Rating Scale, *DSM-IV* Diagnostic and Statistical Manual of Mental Disorders 4th Edition, *Hyp-Imp* Hyperactivity-Impulsivity

### Control group vs. ADHD and simulation group

The ADHD group scored significantly higher than controls on all CAARS and WFIRS subscales. The effect sizes seen here are all considered positive and large (see Table 3) except on WFIRS RISK, which is positive and medium in size. Simulators were found to have significantly higher scores compared to controls on all CAARS and WFIRS subscales. All of these effect sizes represent positive large effects (see Table 3).

### Associations between estimation accuracy scores and descriptive and clinical variables

The total correlation matrix is shown in Table 4. In contrast to the previous analyses, only positive difference scores were included in this analysis. A positive correlation in this context indicates that the variable in the analysis is associated to more negative perceptions (overestimation of impairment), whereas a negative correlation indicates more positive perceptions (underestimation of impairment). A negative correlation in this context was taken as an indication of an association which results in a reduction in inaccuracy. A significant negative correlation was found between CURRENT ADHD SYMPTOMS and ( +)CAARS ACCURACY IMPULSIVITY which was medium in size. Additionally, RETROSPECTIVE ADHD SYMPTOMS was found to be negatively correlated with ( +)CAARS ACCURACY DSM-IV INATTENTION and ( +)CAARS ACCURACY DSM-IV TOTAL, both representing small effect sizes. In the case of ADHD KNOWLEDGE, it was found to be significantly positively correlated with ( +)WFIRS ACCURACY SCHOOL to a small effect size.

**Table 4:** Bivariate Correlations of all positive scores on CAARS and WFIRS accuracy variables with CURRENT ADHD SYMPTOMS (1), RETROSPECTIVE ADHD SYMPTOMS (2), ADHD KNOWLEDGE (3), IQ (4), YEARS OF EDUCATION (5), and AGE (6)

Variable	*N* range	1	2	3	4	5	6
( +)CAARS ACCURACY INATTENTION	48–51	0.079	-0.04	0.041	0.056	− 0.57	−0.158
( +)CAARS ACCURACY HYPERACTIVITY	92–96	0.023	− 0.141	0.025	− 0.041	− 0.121	− 0.068
( +)CAARS ACCURACY IMPULSIVITY	68–70	− 0.311*	− 0.093	0.099	0.018	− 0.027	− 0.111
( +)CAARS ACCURACY SELF-CONCEPT	23–25	0.021	− 0.223	− 0.110	− 0.278	0.339	0.116
( +)CAARS ACCURACY DSM-IV INATTENTION	60–63	− 0.016	− 0.123*	0.048	0.12	− 0.026	0.021
( +)CAARS ACCURACY DSM-IV HYP-IMP	92–96	− 0.136	− 0.17	0.057	− 0.091	− 0.064	− 0.037
( +)CAARS ACCURACY DSM-IV TOTAL	82–86	− 0.032	− 0.277*	0.112	0.08	0.01	− 0.005
( +)WFIRS ACCURACY FAMILY	76–80	−0.068	−0.111	0.015	0.004	−0.089	0.165
( +)WFIRS ACCURACY WORK	87–91	−0.128	−0.176	−0.036	0.040	− 0.095	0.038
( +)WFIRS ACCURACY SCHOOL	85–89	− 0.092	− 0.114	0.257*	− 0.071	− 0.015	0.070
( +)WFIRS ACCURACY LIFE SKILLS	61–65	− 0.064	− 0.097	− 0.002	0.088	0.111	0.097
( +)WFIRS ACCURACY SELF-CONCEPT	29–31	− 0.339	− .192	0.072	0.360	0.187	0.285
( +)WFIRS ACCURACY SOCIAL	70–73	− 0.059	− 0.174	0.045	− 0.027	− 0.125	− 0.082
( +)WFIRS ACCURACY RISK	82–86	− 0.016	− 0.038	0.03	0.137	0.068	0.134

Positive correlations here indicate an association with more pronounced stigma, whereas negative correlations indicate an association with a reduction in stigma. 1 = CURRENT ADHD SYMPTOMS. 2 = RETROSPECTIVE ADHD SYMPTOMS. 3 = ADHD KNOWLEDGE. 4 = IQ. 5 = YEARS OF EDUCATION. 6 = AGE. * = significant effect at the 0.05 level. CAARS = Conner’s Adult ADHD Rating Scales. WFIRS = Weiss Functional Impairment Rating Scale. ( +) = only positive accuracy scores on that subscale. DSM-IV = Diagnostic and Statistical Manual of Mental Disorders 4th Edition. HYP-IMP = Hyperactivity-Impulsivity. ADHD = attention-deficit hyperactivity disorder

Current ADHD symptoms had a small negative correlation with ( +)CAARS ACCURACY DSM-IV HYPERACTIVITY-IMPULSIVITY, ( +)WFIRS ACCURACY WORK, and a medium negative correlation with ( +)CAARS ACCURACY IMPULSIVITY and ( +)WFIRS ACCURACY SELF-CONCEPT. Retrospective ADHD symptoms were found to have a small negative correlation with ( +)CAARS ACCURACY HYPERACTIVITY, ( +)CAARS ACCURACY SELF-CONCEPT, ( +)CAARS ACCURACY DSM-IV INATTENTION, ( +)CAARS DSM-IV HYPERACTIVITY-IMPULSIVITY, ( +)CAARS ACCURACY DSM-IV TOTAL, ( +)WFIRS ACCURACY FAMILY, ( +)WFIRS ACCURACY WORK, ( +)WFIRS ACCURACY SCHOOL, ( +)WFIRS ACCURACY SELF-CONCEPT, and ( +)WFIRS ACCURACY SOCIAL. For all remaining variables, there were negligible negative correlations.

For ADHD knowledge, a small negative correlation was found with ( +)CAARS ACCURACY SELF-CONCEPT, and a small positive correlation with ( +)CAARS ACCURACY DSM-IV TOTAL and ( +)WFIRS ACCURACY SCHOOL. Additionally, except for ( +)CAARS ACCURACY SELF-CONCEPT, ADHD knowledge was found to have a negligible positive correlation with all remaining ( +)CAARS subscales. IQ was found to have a small negative correlation with ( +)CAARS ACCURACY SELF-CONCEPT, a small positive correlation with ( +)CAARS ACCURACY DSM-IV INATTENTION and ( +)WFIRS ACCURACY RISK, and a medium positive correlation with ( +)WFIRS ACCURACY SELF-CONCEPT. In the case of YEARS OF EDUCATION, a small negative correlation was found with ( +)CAARS ACCURACY HYPERACTIVITY and ( +)WFIRS ACCURACY SOCIAL, a small positive correlation with ( +)WFIRS ACCURACY LIFE SKILLS and ( +)WFIRS ACCURACY SELF-CONCEPT, a medium positive correlation with ( +)CAARS ACCURACY SELF-CONCEPT, and a large negative correlation with ( +)CAARS ACCURACY INATTENTION. Age was found to have a small negative correlation with ( +)CAARS ACCURACY INATTENTION and ( +)CAARS IMPULSIVITY, and a small positive correlation with ( +)CAARS ACCURACY SELF-CONCEPT, ( +)WFIRS ACCURACY FAMILY, ( +)WFIRS ACCURACY SELF-CONCEPT, and ( +)WFIRS ACCURACY RISK.

## Discussion

The present study sought to examine the state of public perceptions towards adult ADHD in a number of functional domains, and whether ADHD knowledge or proximity to ADHD symptoms contributed to more accurate perceptions towards individuals with adult ADHD. This was done using a method intended to measure perceptions at a covert, rather than overt level. Analysis revealed that the public perception of adult ADHD was largely negative, and it most closely represented public stigma (Corrigan and Shapiro 2010).

Group comparisons revealed large differences between the ADHD group, simulation group, and control group. The differences between healthy individuals and the ADHD group were evidenced by significant effects of large size across both the CAARS and WFIRS domains. This was as expected, given the breadth of literature showing differences in ADHD symptom domains between individuals with ADHD, and those without (Polancyzk et al. 2014). Significant effects were also found between the simulation group and the control group. However, the effect sizes of these differences were for the majority larger (except in the domain of inattention, DSM-IV inattention, DSM-IV total, and self-concept) across the CAARS and WFIRS domains when compared with the differences found between the ADHD group and control group. This is evidenced that the simulators took their role seriously, and supports the internal validity of the study.

Simulators showed negative misconceptions as seen by statistically significant overestimation of impairments in adult ADHD on the CAARS and WFIRS subscales. The domains of hyperactivity, DSM-IV hyperactivity-impulsivity, DSM-IV total, work, school, and risk were all overestimated to a significantly large effect when compared to the ADHD group. The domains of family and social were significantly overestimated to a medium effect when compared to the ADHD group. Despite not being statistically significant, the simulation group still overestimated impairments to a small effect in impulsivity and DSM-IV inattention when compared to the ADHD group. Surprisingly, impairments in the domain of self-concept were shown to be significantly underestimated by the simulation group to a large effect in the CAARS, and a small effect size in the WFIRS subscales. Both Figs. 1 and 2 show the breadth of domains in which impairments were overestimated. Interestingly, almost all domains (except inattention and self-concept) showed overestimation.

These findings are in contrast with the findings of Mueller et al. (2012) which reported an overall low to moderate levels of stigma using a questionnaire designed specifically to measure public beliefs and perceptions towards adult ADHD. This represents an interesting point of distinction between this study and that of Mueller et al. (2012). The present research measured perceptions implicitly—by having people behave as they imagine someone with adult ADHD would, which gave us insight into their perceptions of ADHD. This varies from the more explicit method used by Mueller et al. which directly asked participants to respond to statements related to measures of stigma (2012). Given the apparent lack of covert rather than overt stigma reduction when compared to levels in the past in the public towards mental health, this new method of measuring public perceptions may offer the opportunity to more accurately measure stigma prevalence, along with specifying in which domains it is most present (Schomerus et al. 2012). It may be possible that measuring a sensitive topic, such as stigmatization, is more validly measured by implicit means, as it may also circumvent any potential social desirability bias (Fisher 1993; Gray 2002). These findings are similar to those of Canu et al. (2008) which found that healthy individuals stigmatize individuals with ADHD, and they did so through questionnaires explicitly measuring participants’ beliefs. This is also consistent with the findings of Eisenberg and Schneider (2007), who demonstrated stigma towards the academic abilities of children with ADHD as shown by the negative estimations of parents and teachers. Taking the negative perceptions towards ADHD found by Canu et al. (2008) and Eisenberg and Schneider (2007) may also be an indication of convergent evidence for the presence of public stigma towards ADHD.

Given the domains in which impairment was most overestimated, it seems these estimations by the simulation group may be more aligned with childhood ADHD than adult ADHD (Masuch et al. 2019). The more overt symptoms of ADHD (hyperactivity, impulsivity, impairments at work, school, etc.) tended to be overestimated the most, whereas covert symptoms like impairments in self-concept were significantly underestimated. The sentiment of ADHD prevalence being dominated by young boys with predominantly hyperactive symptoms has been documented previously and appears to be ratified here (Mueller et al. 2012). This could represent a lack of knowledge in the general population as to the neurodevelopmental nature of ADHD (Biederman 2005). Additionally, this seems to ratify the findings of Ray and Hinnant (2009), which found that ADHD, when depicted in popular media, focused overwhelmingly on childhood ADHD. This particular stigmatization could lead to problems in multiple facets of an individual with adult ADHD’s life, such as education, social, housing prospects, healthcare, and employment (Lebowitz 2016). With individuals already facing the stigma generated by people questioning the legitimacy of ADHD as a disorder, a further layer may be added in that people with ADHD must defend the legitimacy of their particular subtype (Sciutto and Eisenberg 2007).

It was believed that greater knowledge of adult ADHD would be associated with more accurate perceptions of adult ADHD. This was not found to be the case, as knowledge of adult ADHD was significantly associated with more accurate perceptions on just the domain of school. This is contrary to the prevailing belief that increasing knowledge leads to a reduction in stigma (Kosyluk, 2016). However, evidence of ADHD as displayed by the ADHD Knowledge Questionnaire was not the only type of knowledge posited to potentially improve perception accuracy. It was theorized that proximity to ADHD symptomology (as measured by current and retrospective ADHD symptoms) would lead to more accurate perceptions. The current study found retrospective ADHD symptoms had negligible-small associations with more accurate perceptions on all domains. This trend of greater perception accuracy could be an indication of an association between an individuals’ past experiential proximity to ADHD and an increase in their accuracy in estimating impairments. A similar trend of greater perception accuracy was found for current ADHD symptoms, as evidenced by negligible-small associations with all domains except for inattention, hyperactivity, and self-concept. Taken together, these findings may indicate current and retrospective symptomology in an individual to be an area suitable for further investigation. It should be highlighted that these associations were mostly non-significant and small in size, as such their potential relevance should be considered with caution. However, they do lead to some prospectively valuable directions for future research. Given the greater number of associations of ADHD-related symptomology over ADHD-related knowledge, it seems that rather than theoretical knowledge being associated to more accurate perceptions, it may be the experiences of the individual which holds more influence. Additionally, an individual who has more experience of ADHD symptoms, could potentially have unique insight into the more covert symptoms of ADHD, symptoms which may be harder to convey through theoretical methods. However, these findings should be taken as speculatory, as they are based on associations which were predominantly small and non-significant.

## Limitations and future directions

This study was not without its limitations. One potential limitation of this study was a lack of representativeness among the ADHD subtypes in the ADHD group. This is not surprising, however, as research has shown the more overt symptoms of hyperactivity/impulsivity tend to dissipate upon adulthood (Biederman 2005). Nevertheless, the lack of specifically hyperactive-impulsive subtype may have led to a biasing of representation towards symptoms characteristic of the combined and inattentive subtypes. For example, given the overrepresentation of combined/inattentive subtype participants, it stands to reason that impairments in hyperactivity and impulsivity will be overestimated, as while they may be characteristics one thinks of when thinking of ADHD, they were not as prevalent in this particular group. As such, overestimation of impairments in these domains may be exaggerated and not conducive to a more heterogeneous ADHD population. Adding to this, the measure of actual impairment in a domain was calculated using the average scores of the ADHD group in said domain. This does not account for individual variation. This is especially important given the variable nature between ADHD subtypes. As such, some of the averages may favour overrepresented subtypes in our sample. A possible solution in future research could be to ensure that the sample is more heterogeneous in this domain at the recruiting stage of the study.

Furthermore, it may be that the approach to measuring ADHD knowledge was not valid. ADHD knowledge was estimated using the ADHD Knowledge Questionnaire, which gives a total score as an aggregate of the different domains it encompasses (Gaastra et al. 2015). It is possible that using this single score, there is some information lost for the sake of generalizability. However, when examining the distribution of knowledge scores across the simulation group, they appear to be well distributed. Future studies may consider measuring knowledge using more than one method to improve validity.

Likewise, it may be that the difference in characteristics between the ADHD group and the simulation group represents a threat to the rigor of the study. This was visible as the ADHD group having significantly higher age and lower years of education when compared to the simulation group. This may have resulted in an overrepresentation of covert symptoms, given the neurodevelopmental nature of the disorder. This could have had the effect of over/underestimation of impairments as a result of more intense covert symptoms. For example, this may be the reason for impairments in the domain of self-concept being significantly underestimated by simulators.

Additionally, it is possible that simulators did not make their best effort to feign ADHD. The Effort Scale was used to combat this possibility; however, it could have proved confounding to the study if a participant did not respond to it truthfully. A more rigorous examination of participants’ effort may be necessary in the future to safeguard internal validity. The Effort Scale could be expanded to include multiple measures of effort with the intention of estimating an aggregate of a participant’s effort.

This study was largely based on examining the overestimations of impairments, and not the underestimations of impairments. This was done because the focus of this study was on perceptions relating to public stigma, which was represented by overestimations of impairment. An individual who may have doubts as to the legitimacy of ADHD as a disorder may indicate that the individual has exaggerated or fabricated their symptoms (Sciutto and Eisenberg 2007). This could result in a participant underestimating impairments in ADHD, which would be taken as a positive representation of ADHD by the conceptualization of stigmatization used in this study, when in fact, it originates from a negative belief. However, the underestimations by participants represent important information in its own right. These could be taken as a pseudo-opposite of stigmatization. Future studies may look at what are the associations with underestimation of impairment, and whether theoretical or contact-based education is associated with a reduction or increase in underestimation.

Likewise, another potential aspect which this research is missing is capturing the overt expressions of public perceptions. By measuring overt expressions in conjunction with covert expressions, it may be possible to increase the validity of this new method. Not only this, but it may serve to illustrate a more complete conceptualization of a participant’s perception towards ADHD. This may also facilitate comparisons of consistency between a participants overt and covert perceptions on a single topic. In doing so, it may be possible to estimate to what extent does the previously mentioned social desirability bias influences a participant’s responses (Fisher 1993; Gray 2002). Future studies should consider combining this new method of measuring covert perceptions, with another method to measure overt perceptions, for example, the ADHD Stigma Questionnaire (Kellison et al. 2010).

As previously stated, this study used a new method to measure perceptions. While this brings novelty to the research, it also brings uncertainty. Ideally, this research would have included a measure of validity to test the extent to which the construct of interest was being accurately estimated. In order for the validity of this new method to be proven in more detail, more research is needed attempting to assess the convergent validity of the method. Until this validity has been proven in greater detail, the results of the present research should be considered preliminary.

Another important factor to be considered is sample size. Following the removal of participants based on exclusion criteria, the total sample size was 310 (98 participants with ADHD, 222 participants not diagnosed with ADHD). This number of participants may be considered lacking when trying to generalize the findings to accurately represent populations. This could lead to increased variability within the results, potentially decreasing the external validity of the research. This is not the only benefit of a larger sample size. By increasing the sample size, it becomes more feasible to investigate the effects of descriptive characteristics within the sample, for example, age, gender, and education. Furthermore, clinical characteristics could potentially be explored, such as comorbidities, treatment outcomes, and subtype comparisons. This would allow for many possibilities of interest which could not be considered given the current sample size. As such, future studies should put a greater emphasis on gathering a larger number of participants to ensure that validity is safeguarded.

Finally, this study can be seen as limited in its scope of potential scenarios to have simulators feigning. Further studies should examine the aspects of life in which an individual with ADHD may experience stigmatization, for example in education, social settings, housing prospects, healthcare, and employment (Lebowitz 2016). Studies may design scenarios which depict these situations. This could be done by, for example, having individuals feign ADHD in a fictitious job or housing interview. By doing this, it may be possible to deconstruct the situations in which individuals with ADHD experience stigma the most, and pinpoint which factors contribute toward the stigmatization. Additionally, further studies may focus on expanding the breadth of stigma research across more ADHD-related scales than just the CAARS and WFIRS. Additionally, future research may investigate perceptions towards both adult and child ADHD across the same scale of impairment. This could offer insight into the different ways stigmatization presents itself across a neurodevelopmental disorder.

## Conclusion

Perceptions indicative of public stigma were demonstrated in the vast majority of domains of interest. Specifically, it was the more overt symptom domains which were found to be misconceived the most: hyperactivity, DSM-IV hyperactivity-impulsivity, DSM-IV total, work, school, and risk. Moreover, as the simulators overestimated the overt symptom impairments the most, it is possible that the conceptualization of ADHD in the public view is dominated by that of childhood ADHD. Additionally, increased knowledge of ADHD was not found to be greatly associated with more accurate estimations of impairments, however, increased current and retrospective symptoms experienced by the participants were found to be associated with more accurate estimations in a number of domains. These associations were small in size, however, and as such should be taken as an area which may lead to interesting future research. It may also open a discussion as to whether it is possible that more experiential knowledge will potentially be more effective at increasing public understanding of adult ADHD than theoretical means.

## Data Availability

Yes—all data are fully available without restriction.
